# Characteristics of repair tissue in second-look and third-look biopsies from patients treated with engineered cartilage: relationship to symptomatology and time after implantation

**DOI:** 10.1186/ar2549

**Published:** 2008-11-11

**Authors:** Paola Brun, Sally C Dickinson, Barbara Zavan, Roberta Cortivo, Anthony P Hollander, Giovanni Abatangelo

**Affiliations:** 1 Department of Histology, Microbiology and Medical Biotechnology, Histology Unit, Faculty of Medicine, University of Padova, Viale G. Colombo 3, 35121 Padova, Italy; 2 Department of Cellular & Molecular Medicine, University of Bristol, School of Medicine Sciences, University Walk, Bristol BS8 1TD, UK

## Abstract

**Introduction:**

The present study established characteristics of tissue regrowth in patients suffering knee lesions treated with grafts of autologous chondrocytes grown on three-dimensional hyaluronic acid biomaterials.

**Methods:**

This multicentred study involved a second-look arthroscopy/biopsy, 5 to 33 months post implant (*n* = 63). Seven patients allowed a third-look biopsy, three of which were performed 18 months post implant. Characteristics of tissues were histologically and histochemically evaluated. The remaining bone stubs were evaluated for cartilage/bone integration. For data analysis, biopsies were further divided into those obtained from postoperative symptomatic patients (*n* = 41) or from asymptomatic patients (*n* = 22).

**Results:**

The percentage of hyaline regenerated tissues was significantly greater in biopsies obtained after, versus within, 18 months of implantation. Differences were also observed between symptomatic and asymptomatic patients: reparative tissues taken from symptomatic patients 18 months after grafting were mainly fibrocartilage or mixed (hyaline–fibrocartilage) tissue, while tissues taken from asymptomatic patients were hyaline cartilage in 83% of biopsies. In a small group of asymptomatic patients (*n* = 3), second-look and third-look biopsies taken 18 months after surgery confirmed maturation of the newly formed tissue over time. Cartilage maturation occurred from the inner regions of the graft, in contact with subchondral bone, towards the periphery of the implant.

**Conclusions:**

The study indicates that, in asymptomatic patients after chondrocyte implantation, regenerated tissue undergoes a process of maturation that in the majority of cases takes longer than 18 months for completion and leads to hyaline tissue and not fibrous cartilage. Persistence of symptoms might reflect the presence of a nonhyaline cartilage repair tissue.

## Introduction

Full-thickness cartilage defects do not heal spontaneously. Lesions that penetrate the subchondral bone undergo repair with fibrocartilage, a tissue that resists tension but not compression [1,2]. Current therapies, such as transplantation of healthy cartilage, microfracture of the subchondral bone plate and implantation of artificial polymers or metal prostheses, have many limitations [3,4]. In past decades, investigators have pursued techniques for stimulating articular repair and regeneration. In particular, autologous chondrocyte implantation is a promising cell therapy technique [5-8] – yet it is limited by the complexity of the surgical procedure required for periosteal harvesting and by its associated morbidity.

A more recent approach to treating cartilage defects is the use of *in vitro *engineered tissue obtained using autologous chondrocytes seeded and cultured onto biodegradable and biocompatible scaffolds. Three-dimensional biodegradable materials derived from the total esterification of hyaluronan with benzyl alcohol and constructed with a nonwoven configuration (Hyaff-11) have been successfully utilized for culturing autologous chondrocytes [9-12]. Human articular chondrocytes derived from knee articular biopsies and cultured as a monolayer de-differentiate after a few days of cell expansion, but a three-dimensional environment promotes re-differentiation to the cartilage phenotype. RNA analysis and immunohistochemistry reveal that cells sustain the expression of cartilage genes and proteins, such as collagen type II, type IX, and type X, aggrecan and sox 9 [13,14]. Clinical medium-term results on the treatment of 0.5 to 15 cm^2 ^defects with tissue engineered grafts made ofautologous chondrocytes grown on Hyaff 11 (Hyalograft^® ^C Autograft; Fidia Advanced Biopolymers, Abano Terme, Italy) were very favourable, with more than 90% of patients expressing an improvement in knee symptoms and function and a very limited number of complications [15,16]. The Hyalograft^® ^C Autograft was simply placed into the prepared lesion, where it was stable and without the need for any fixation method. The extent of tissue regeneration after Hyalograft^® ^C Autograft implantation was also studied in 23 patients using a new quantitative analysis of collagen type II that distinguishes hyaline cartilage from the type I collagen found predominantly in fibrocartilage [17,18]. In that study, tissue engineered grafts induced cartilage regeneration as early as 11 months after implantation, and integration of the newly formed tissues with underlying bone was good in all patients.

In the present study, we analysed 70 biopsies, 63 second-look biopsies and seven third-look biopsies, taken from patients 5 to 33 months after Hyalograft^® ^C Autograft implantation for cartilage lesions of the knee. Biopsies were divided between symptomatic and asymptomatic patients to identify the relationship between reconstructive success and histological composition of the new tissue. The analysis of cartilage maturation 18 months post implant in the same individual, however, was only possible in three patients.

## Materials and methods

### Patients

The samples analysed were from a series of consecutive biopsies received at our laboratory from 63 patients with knee cartilage lesions treated with Hyalograft^® ^C Autograft at 11 Italian orthopaedic centres. For each centre, the local ethical committee approved the study; after informed consent, biopsies were sampled from patients at the time of their follow-up arthroscopy. Patients' sole inclusion criterion into the study was their written informed consent to undergo a biopsy at the time of arthroscopy. No exclusion criteria were applied.

### Tissue engineering and the Hyalograft^® ^C surgical technique

The Hyalograft^® ^C Autograft is a tissue graft consisting of autologous chondrocytes grown on a three-dimensional scaffold made of hyaluronic acid, used in clinical practice since 1999 for the treatment of full-thickness cartilage defects. It is obtained by seeding autologous chondrocytes into a three-dimensional biodegradable material derived from the total esterification of hyaluronan with benzyl alcohol and constructed with a non-woven configuration (Hyaff 11). Briefly, cells were obtained from the digestion of biopsies as described elsewhere [11], were re-suspended in DMEM medium, and were seeded and cultured onto the Hyaff 11 scaffold for 14 days.

In the majority of cases, the Hyalograft^® ^C Autograft was positioned at the lesion site by a mini-arthrotomy using a simple procedure. In the case of large uncontained defects, particularly in the patello-femoral compartment, fibrin glue and/or sutures were used to keep the graft in place. Subsequent immobilization was recommended for 12 to 24 hours post grafting.

### Second-look and third-look biopsy harvesting

Seventy biopsies from 63 patients treated with tissue-engineered cartilage were analysed. All patients had the second-look biopsy at the time of their follow-up arthroscopy, and seven patients permitted a third-look biopsy at the time of their second arthroscopy. These subsequent biopsies were taken presumably from the same location. The biopsies (2 mm diameter) were taken primarily (*n* = 62) from the medial or lateral condyle; others were taken from the tibial plate (*n* = 2), trochlea (*n* = 1) or patella (*n* = 5).

### Histological evaluation

Full-thickness cylindrical biopsies with a 2 mm diameter extending from the articular surface to the subchondral bone were obtained from the centre of the defect, were embedded in optimal cutting temperature (OCT) embedding medium, were snap-frozen and were cut into 7 μm sections. Cellular and histochemical characteristics of the repair tissue were evaluated histologically and immunohistochemically.

For routine histology, specimens were stained with H & E to visualize the cellularity, the morphology of cells and the matrix appearance of the repair tissue. Safranin-O stain was used to detect the presence of glycosaminoglycans. Specimens were also analysed using polarized light microscopy to examine collagen organization in the tissue extracellular matrix.

For immunohistological analysis with specific antibodies – monoclonal collagen I antibody (Sigma, St Louis, MO, USA) and monoclonal anti-collagen II antibody (Developmental Studies Hybridoma Bank, Iowa City, IA, USA) – frozen sections were fixed in acetone, were predigested with hyaluronidase (to expose collagen epitopes to antibodies) and were subsequently incubated in Tris buffer saline (10 mM Tris, 150 mM NaCl, pH 7.4) containing 10% normal rabbit serum (Dako, Glostrup, Denmark), primary antibody and secondary rabbit anti-mouse antibody (Dako), and were finally treated with an alkaline phosphatise-antialkaline phosphatise (APAAP) complex (Dako) and stained with fast red (Sigma).

Human placenta was used as a positive control for collagen type I, and healthy articular cartilage for collagen type II. Negative control slides were obtained by treating specimens with mouse monoclonal IgG_1 _or mouse monoclonal IgM (Dako). The positive red reaction for collagens obtained with immunohistochemistry was classified as very intense, intense, slight or absent.

Repair tissue was evaluated with specific histological criteria regarding cellularity, the cell distribution and the matrix composition in hyaline tissue, fibrocartilage and mixed tissues. Two investigators scored each biopsy under blind conditions, and classified them: as hyaline-like cartilage when round cells in lacunae were in clusters and columns, and collagen fibrils, mainly of type II collagen (very intense), were parallel to the surface; as fibrocartilage when cells with rounded morphology and collagen type I fibrils (very intense) were localized randomly; or as mixed tissues when hyaline-like tissue and fibrocartilage-like tissue were present, with a moderate content of collagen type II (intense).

The biopsies were divided into two groups considering the time from implantation: biopsies taken within 18 months of Hyalograft^® ^C Autograft implantation, and biopsies taken longer than 18 months after surgery.

### Analysis of tidemark

The degree of integration of implanted tissue to bone was analysed by the presence of a tidemark; that is, the calcified interface between cartilage and bone where the tissues have become mineralized. This analysis was only performed on samples containing subchondral bone (21 cases). In such cases, the bone was separated from the overlying cartilage, decalcified and placed in paraffin. The samples then underwent histological analysis (H & E) to assess the degree of integration between the newly formed cartilage and the underlying bone surface.

Stained sections were independently scored by three investigators, blinded to the treatment outcome, each describing the histological appearance under light microscopy (Zeiss, Oberkochen, Germany). The presence of a tidemark (yes or no) was also evaluated.

### Statistical analyses

The Fisher exact test was used to compare the morphology of regenerated tissues between the two treated groups of patients (biopsies obtained longer than 18 months after surgery and biopsies obtained within 18 months of surgery). *P *< 0.05 was considered significant. No intrapatient analysis of results over time was performed due to the small number of patients (*n* = 3) for whom this comparison was appropriate.

## Results

### Patients

Baseline clinical data are presented in Table 1. Seventy biopsies from 63 patients treated with tissue-engineered cartilage were analysed. Of these, 47 biopsies (67.1%) were harvested from asymptomatic patients who had agreed to biopsy harvesting, despite the fact that they no longer suffered symptoms. The other biopsies (*n* = 23, 32.9%) were from symptomatic patients who were biopsied for a wide variability of clinical reasons, such as knee pain, fibrillation, gonalgia, swelling or other symptoms. There were 22 patients who reported symptoms (34.9%; one of them had a third-look biopsy) and 41 patients who had no symptom at all (65.1%; six of them had a third-look biopsy).

**Table 1:** Characteristics of patients (*n* = 63) suffering knee lesions who were treated with a tissue engineered graft made with autologous chondrocytes grown on a three-dimensional hyaluronic acid-based biomaterial

Characteristic	Baseline
Age (years)	
∘ Mean (standard deviation)	39 (11.47)
∘ Range (minimum to maximum)	16 to 64
Gender (male/female)	41/22
Biopsies from asymptomatic patients	23 biopsies
Biopsies from asymptomatic patients	47 biopsies
Single lesion	39 patients
Multiple lesions	31 patients
Location of defect	
∘ Medial or lateral condyle	62 biopsies
∘ Tibial plateau	2 biopsies
∘ Trochlea	1 biopsy
∘ Patella	5 biopsies
Total surface area for all patients (mean (standard deviation))	
∘ Range in asymptomatic patients (cm^2^)	5.2 (2.9)
∘ Range in symptomatic patients (cm^2^)	3.4 (2.8)
Outerbridge grade	
∘ Outerbridge grade IV	57 patients
∘ Outerbridge grade III	6 patients

Biopsies (*n* = 70) were classified by their time after implantation: before 18 months of follow-up or after longer than 18 months of follow-up.

The patient cohort included 41 men and 22 women with a mean age of 39 years (standard deviation = 11.47; minimum age = 16 years, maximum age = 64 years). The mean follow-up time between the application of Hyalograft^®^C and the biopsy was 14.1 months (minimum time = 5 months, maximum time = 33 months). The mean lesion area in treated asymptomatic patients (5.2 cm^2^, standard deviation = 2.9) was greater than the lesion area of symptomatic patients (3.4 cm^2^, standard deviation = 2.8) (see Table 1).

### Histological and immunohistochemical analysis

**Figure 1:**
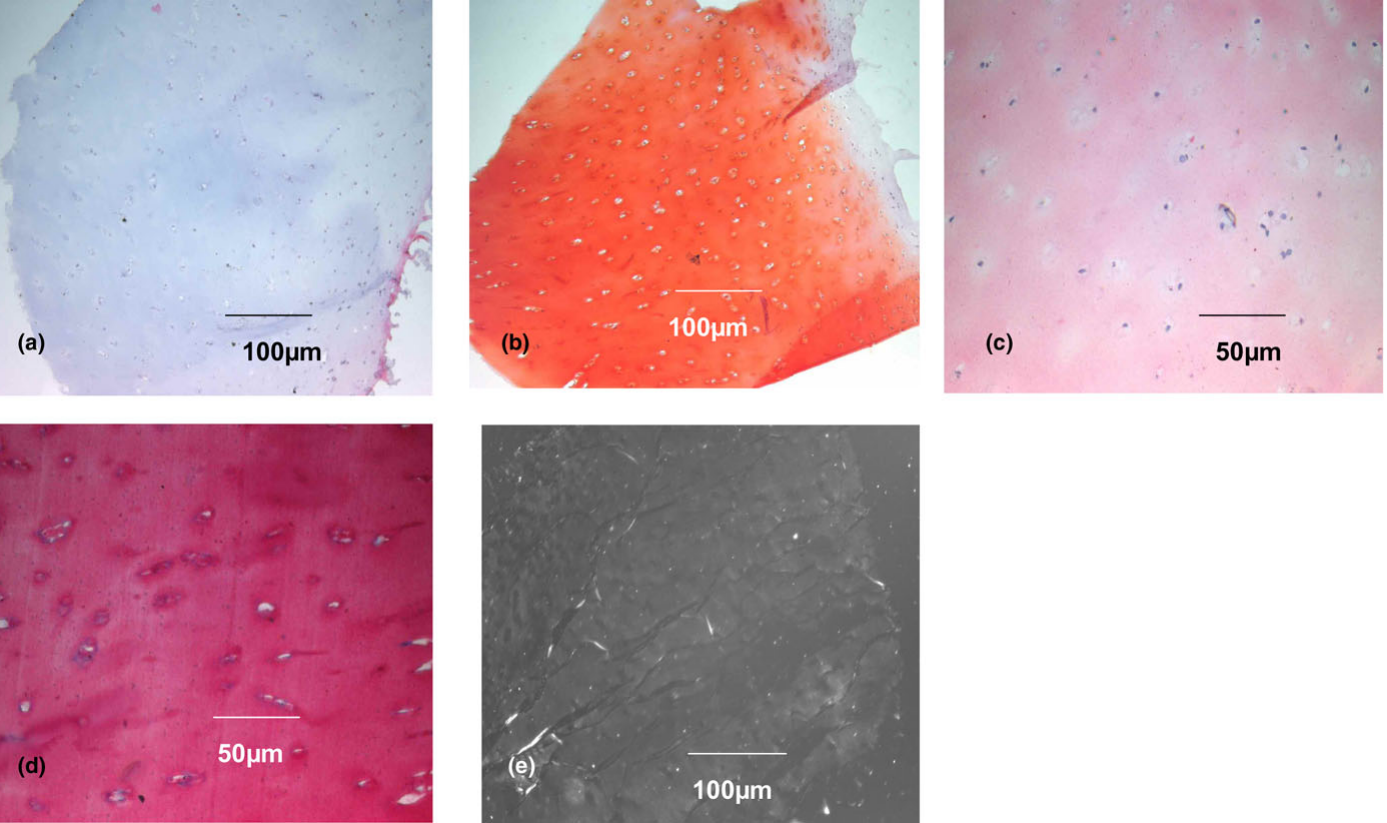
Histological and immunohistochemical analysis of a second-look cartilage biopsy with hyaline characteristics. Analysis of a second-look cartilage biopsy with hyaline characteristics taken 14 months after Hyalograft^® ^C Autograft implantation. **(a) **H & E and eosin staining. **(b) **Glycosaminoglycan (safranin-O) staining. **(c) **Collagen type I (immunohistochemistry). **(d) **Collagen type II (immunohistochemistry). **(e) **Polarized light microscopy.

**Figure 2:**
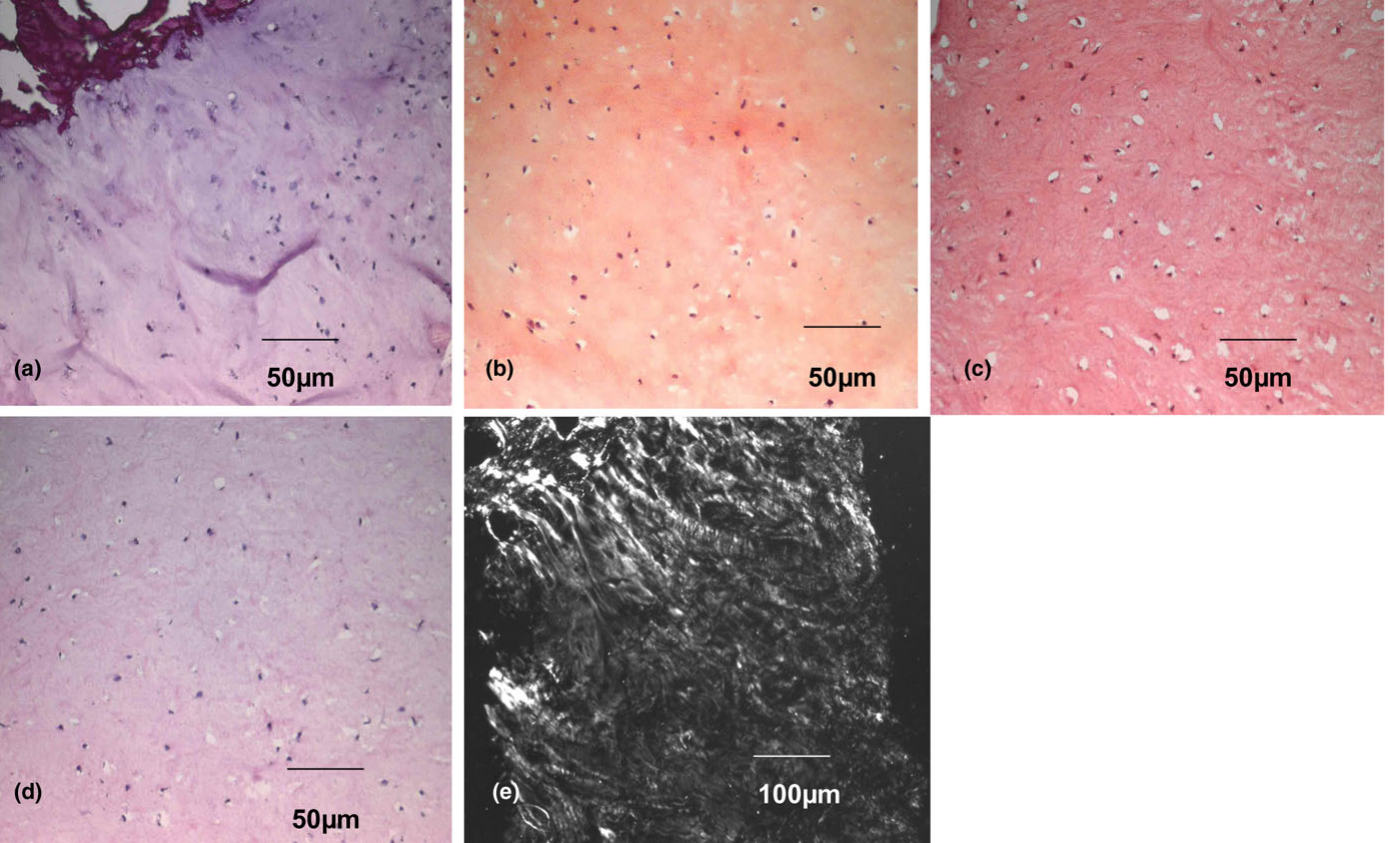
Histological and immunohistochemical analysis of a second-look cartilage biopsy with fibrocartilage characteristics. Analysis of a second-look cartilage biopsy with fibrocartilage characteristics taken 10 months after Hyalograft^® ^C Autograft implantation. **(a) **H & E staining. **(b) **Glycosaminoglycan (safranin-O) staining. **(c) **Collagen type I (immunohistochemistry). **(d) **Collagen type II (immunohistochemistry). **(e) **Polarized light microscopy.

**Figure 3:**
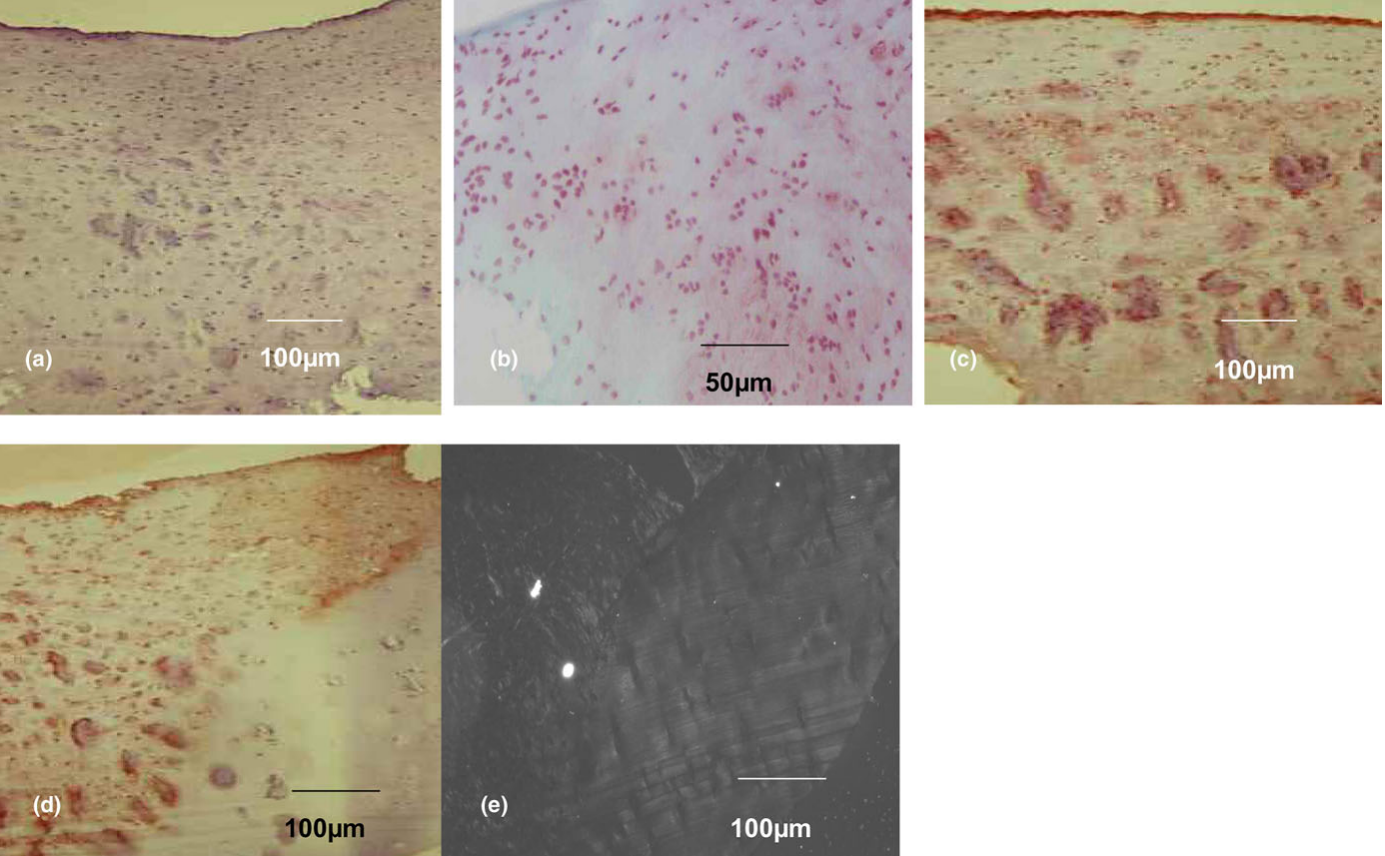
Histological and immunohistochemical analysis of a second-look cartilage biopsy with mixed (hyaline and fibrocartilage) characteristics. Analysis of a second-look cartilage biopsy with mixed characteristics taken 10 months after Hyalograft^® ^C Autograft implantation. **(a) **H & E staining. **(b) **Glycosaminoglycan (safranin-O staining). **(c) **Collagen type I (immunohistochemistry). **(d) **Collagen type II (immunohistochemistry). **(e) **Polarized light microscopy.

When all biopsies were grouped together (*n* = 70), histology showed that 19 biopsies (27.2%) were composed of tissue with a typical hyaline cartilage cell distribution in clusters and columns in the lacunae (Figure 1a,b); that 36 biopsies (51.4%) demonstrated cells with rounded morphology that were localized randomly, as in fibrocartilage (Figure 2a,b); and that 15 biopsies (21.4%) demonstrated a mixed-type tissue (hyaline-like and fibrocartilage-like tissue) (Figure 3a,b). Histochemical analysis revealed that collagen type II was more expressed in hyaline tissue (very intense), whereas collagen type I was predominant in fibrocartilage (very intense) (Figures 1c,d, 2c,d and 3c,d).

**Figure 4:**
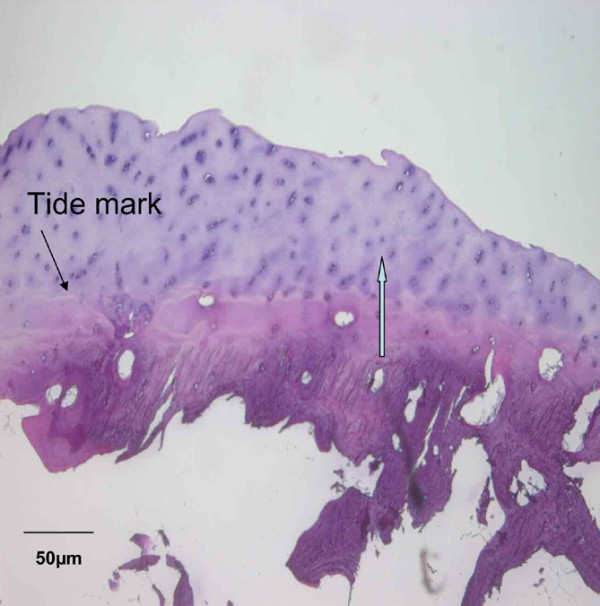
Tide mark evidence for repair tissue. Tidemark evidence from a patient with hyaline repair tissue 12 months after implantation of the Hyalograft^® ^C Autograft. Blue arrow, surface direction.

These data were confirmed by analyses of biopsies with polarized microscopy, which demonstrated the presence of typical hyaline collagen fibrils parallel to the surface of the biopsy with a normal cartilage cell organization. In contrast, biopsies with an abnormal cartilage cell distribution had randomly oriented collagen fibrils, typical of fibrocartilage (Figures 1e, 2e and 3e). In 21 samples, it was also possible to analyse the interface between cartilage and subchondral bone. H & E analysis at their interface showed that all grafted cartilage was well integrated, regardless of whether it was hyaline, fibrocartilage or mixed. Furthermore, a tidemark typical of native cartilage was observed in all biopsies classified as hyaline cartilage (Figure 4).

**Figure 5:**
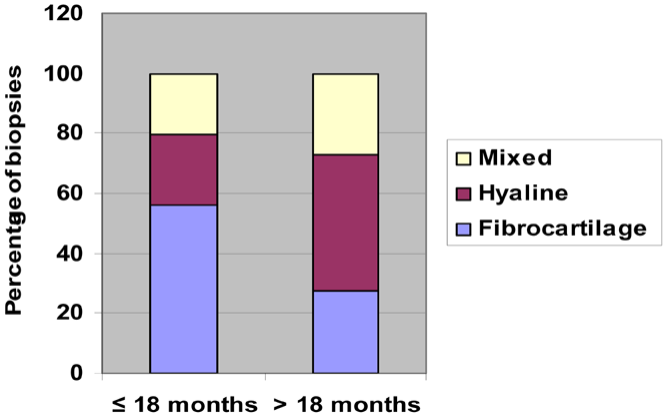
Histological and immunohistochemical analysis data for biopsies obtained before or after 18 months post implantation. Data obtained from histological and immunohistochemical analyses of the biopsies were divided into two groups: biopsies taken before (*n* = 59) or after (*n* = 11) the cutoff point of 18 months post Hyalograft^® ^C Autograft implantation. Only 23.7% of specimens (*n* = 59) obtained from patients before 18 months showed characteristics of hyaline cartilage, whereas 45.4% of specimens (*n* = 11) taken after more than 18 months were of hyaline cartilage. Fibrocartilage was present in 55.9% of biopsies taken before 18 months and in 27.3% of those taken after more than 18 months. Similarly, mixed tissue was present in 20.3% of biopsies taken before 18 months and in 27.3% of those taken after more than 18 months.

The biopsies were then divided into two groups: biopsies obtained within 18 months from Hyalograft^® ^C Autograft implantation, and biopsies obtained longer than 18 months after surgery. The biopsies taken after a longer follow-up period showed a higher percentage of hyaline cartilage (45.4% hyaline cartilage versus 23.7% in biopsies taken within the first 18 months). Fibrocartilage was present in 55.9% of biopsies taken within 18 months after implantation and in 27.3% of those taken after that period. Mixed tissue was present in 20.3% of biopsies taken within 18 months after implantation and in 27.3% of those taken after that period (Figure 5).

**Figure 6:**
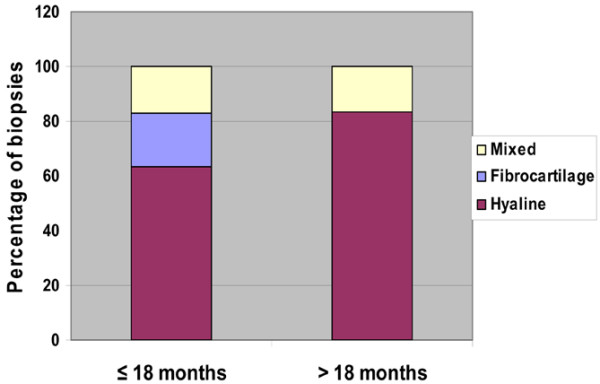
Histological and immunohistochemical analysis data for biopsies obtained from asymptomatic patients only (*n* = 47). The biopsies were divided into those taken before (*n* = 41) or after (*n* = 6) the cutoff point of 18 months post Hyalograft^® ^C Autograft implantation. Six biopsies were taken from asymptomatic patients longer than 18 months after surgery. Results showed that five out of six consisted primarily of hyaline cartilage (83.3%); only one biopsy contained mixed tissue (17.7%).

When biopsies were limited to those from asymptomatic patients taken longer than 18 months after grafting (*n* = 6), the percentage of samples with predominantly hyaline cartilage increased to 83% (Figure 6). Using the Fisher exact test, hyaline tissue was present in significantly greater quantities in biopsies taken longer than 18 months from grafting (*P *= 0.0042).

In biopsies of symptomatic patients taken longer than 18 months after grafting (*n* = 5), reparative tissue was fibrocartilage (60%) or mixed tissue (40%).

When biopsies were limited to only those from asymptomatic patients who had received both second-look and third-look biopsies (*n* = 3), with the third-look biopsies taken longer than 18 months after surgery, we observed clear evidence of cartilage maturation over time. In fact, analysis of these same patients at the second-look biopsies (mean time from implantation = 13.6 months) showed fibrocartilage, mixed tissue and hyaline tissue, while the third-look biopsies (mean time from implantation = 30.6 months) were all hyaline cartilage.

**Figure 7:**
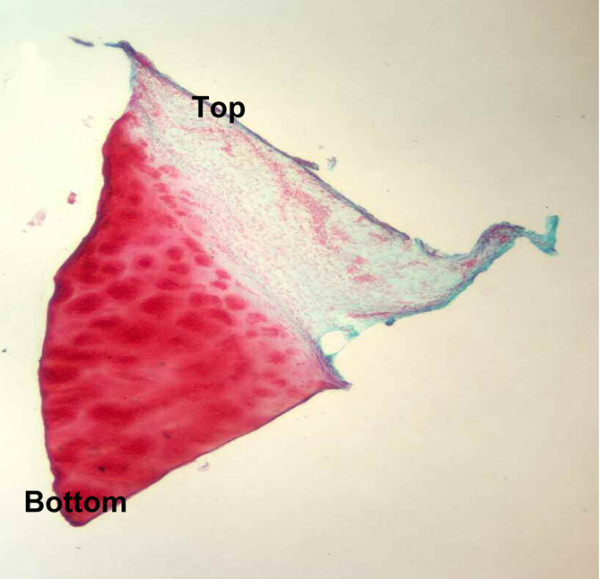
Direction of tissue maturation in a biopsy classified as mixed. Glycosaminoglycan (safranin-O) staining from a patient with mixed tissue 18 months after implantation with Hyalograft^® ^C Autograft.

In most of the specimens classified as mixed, we observed that the direction of maturation was bottom-up. In the inner region of the implant, in contact with the subcondral bone, we noted a hyaline-like tissue; proceeding toward the edges, the regenerated tissue was mostly fibrocartilage (Figure 7), confirmed by analysis of the type I collagen content (data not shown).

## Discussion

In the present study, Hyalograft^® ^C Autografts were implanted for the treatment of cartilage lesions. As previous studies had already demonstrated [18], the clinical outcome of Hyalograft^® ^C Autograft implantation is as good as and comparable with results obtained with autologous chondrocyte implantation (ACI). This technique does not involve open surgery and thus markedly reduces joint trauma compared with ACI. Moreover, in the majority of cases, implantation is stable and does not require any fixation method because of the intrinsic adhesive properties of the hyaluronan scaffold. Consequently, there is no need to harvest a periosteal flap. Other studies have demonstrated that cartilage tissue regeneration after surgery with Hyalograft^® ^C Autograft leads to good results in terms of collagen composition and integration with the subchondral bone [17]. In the present study, the interface between new generated cartilage and subchondral bone was always well integrated, regardless of whether it was hyaline tissue, fibrocartilage or mixed-type tissue.

Characterization of cartilage maturation after Hyalograft^® ^C Autograft implantation demonstrated that cartilage regeneration was a slow process that, in most cases, took longer than 18 months. The most important result of the present study was that the percentage of hyaline cartilage was greater in biopsies obtained longer than 18 months after implantation rather than within 18 months of implantation. Moreover, we noted a correlation between the symptomatology of patients and the nature of the reparative tissue. Asymptomatic patients developed predominantly hyaline tissue in a highly significant percentage of cases, while symptomatic patients presented fibrocartilage or mixed tissue.

These results were confirmed in the small subgroup of three asymptomatic patients who received both second-look and third-look biopsies, with the third-look biopsies taken longer than 18 months after surgery: there was clear evidence of cartilage maturation over time. Second-look biopsies showed fibrocartilage, hyaline tissue or mixed tissue, while third-look biopsies derived from the same patients were all hyaline tissue. The patient number is very limited because patients with a good clinical outcome rarely consent to additional biopsies.

With regard to the direction of tissue regeneration over time, histological observation of specimens demonstrated that, in most mixed tissue, regeneration occurred first in contact with the subchondral bone and then proceeded toward the outer region. Soon after grafting, chondrocytes are known to proliferate and to synthesize extracellular matrix cartilage with a poor molecular network rich in collagen type I – leading to the formation of immature tissue that, in time, differentiates and becomes organized into hyaline tissue with cells in columns immersed in a specialized extracellular matrix rich in collagen type II. The present data indicate that cartilage regeneration might resemble tissue formation during embryogenesis, even if tissue strategies are different from early embryonic events. Previous studies have demonstrated the plasticity of mature chondrocytes and how they de-differentiate towards the phenotypical characteristics of immature chondrocytes when cultured in two dimensions [19]. Other studies have revealed that environmental conditions are very important for cell re-differentiation, and it is well known that the biochemical and physical factors act in concert with genes to mediate their expression [20]. The chemical composition of scaffolds can also be inductive for cell proliferation [21] or for differentiation in adults. In fact, the presence of hyaluronic acid plays an important role in cartilage differentiation, as demonstrated in the embryo [22,23].

From our analysis of second-look and third-look cartilage biopsies from patients with cartilage lesions treated with Hyalograft^® ^C Autograft, we can state that the plasticity of mature chondrocytes is sufficient to enable de-differentiated cells to revert to differentiation. For this reason, mature cells can be used in *in vitro *cartilage tissue reconstruction with impressive results in terms of grafting and clinical outcome. In the three asymptomatic patients who received both second-look and third-look biopsies, with the last biopsy taken 18 months after surgery, cartilage maturation was shown to improve with time. We can therefore affirm that cartilage regeneration is a long process usually taking more than 18 months to be completed.

## Conclusion

Additional investigations will aim to identify the relationship between a good clinical outcome and patient symptomatology. This knowledge would help us to understand how to modulate and optimize natural cartilage turnover in adults, and how to improve the clinical outcome of the engineered cartilage implantation procedure.

## Abbreviations

DMEM: Dulbecco's modified Eagle's medium; H & E: haematoxylin and eosin; Hyalograft^® ^C Autograft: autologous chondrocytes grown on Hyaff 11

## Competing interests

The authors declare that they have no competing interests.

## Authors' contributions

PB carried out the analytical approach, data collection and analysis, and the write up. SCD carried out the data collection and analysis. BZ carried out the data collection and analysis. RC carried out the patient recruitment and application of classification criteria. APH carried out the patient recruitment, the review of the manuscript, and supervision. GA carried out the development of the protocol, the analytical approach, the review of the manuscript and supervision.
